# Assessment of Vestibular Function in Patients with Congenital Bilateral Sensorineural Hearing Loss: A Case-Control Study

**DOI:** 10.3390/jcm15093431

**Published:** 2026-04-30

**Authors:** Michalina Piechocka, Jarosław Markowski, Przemysław Śpiewak, Paweł Dobosz, Sylwia Kopeć-Gołdyn, Marcin Piechocki

**Affiliations:** 1 Department of Otolaryngology and Head and Neck Oncologic Surgery, 5th Military Clinical Hospital with Polyclinic, 30-901 Krakow, Poland; p.dobosz@5wszk.com.pl; 2 Department of Laryngology, Faculty of Medical Sciences in Katowice, Medical University of Silesia, 40-027 Katowice, Poland; jmarkowski@sum.edu.pl; 3 Audiofonika Center for Audiology, Phoniatrics and Otoneurology, 43-300 Bielsko-Biała, Poland; przemyslaw.spiewak@gmail.com (P.Ś.); s.kopec03@gmail.com (S.K.-G.); 4 Doctoral School of Medical and Health Sciences, Jagiellonian University Medical College, 31-530 Krakow, Poland; mpiech98@gmail.com

**Keywords:** congenital sensorineural hearing loss, vestibular dysfunction, vestibulo-ocular reflex, video head impulse test, caloric test, rotational chair test, vestibular assessment, motor development

## Abstract

**Background/Objectives**: The cochlea and vestibular organs develop concurrently during embryogenesis and share anatomical and functional pathways. As a result, congenital factors affecting the vestibulocochlear system may impair both hearing and vestibular function. Despite this, the relationship between congenital bilateral sensorineural hearing loss (SNHL) and vestibular dysfunction remains insufficiently defined. This study evaluated vestibular function in patients with congenital bilateral SNHL and investigated the association between hearing loss severity and vestibular function. **Methods**: A total of 202 participants aged 7–31 years were enrolled, including 102 patients with congenital bilateral SNHL and 100 healthy controls. Vestibular function was assessed using videonystagmography (VNG) during sinusoidal harmonic acceleration (SHA) rotational testing and caloric testing performed according to the Fitzgerald–Hallpike protocol, as well as with the video head impulse test (vHIT). Statistical analyses compared vestibular parameters between groups and assessed correlations with hearing loss severity. **Results**: Patients with congenital bilateral SNHL exhibited significantly lower vestibulo-ocular reflex (VOR) values in the SHA test compared to controls. Greater hearing loss severity was associated with lower VOR gain values. No statistically significant differences were observed between groups in caloric test results or vHIT VOR gain values. However, corrective saccades during vHIT were identified exclusively in patients with hearing loss and occurred in approximately 15% of cases. Furthermore, the age of independent walking was significantly delayed in the study group compared to controls. **Conclusions**: Congenital bilateral SNHL is associated with vestibular dysfunction, as evidenced by abnormal SHA test results and the presence of corrective saccades in vHIT. These patients may also experience delayed motor development. These findings suggest that vestibular dysfunction may be present in patients with congenital sensorineural hearing loss and may have functional implications.

## 1. Introduction

Congenital sensorineural hearing loss (SNHL) is one of the most common sensory impairments in childhood, affecting approximately 1–3 per 1000 live-born infants worldwide [1,2]. Early diagnosis has significantly improved following the implementation of universal newborn hearing screening programs, allowing timely auditory rehabilitation. However, clinical evaluation of patients with congenital hearing loss remains primarily focused on auditory function, while the functional status of the vestibular apparatus is often overlooked.

The cochlea and vestibular organs originate from the otic placode during early embryogenesis and share common anatomical and functional pathways [3]. Consequently, pathological factors affecting the inner ear during fetal development may simultaneously impair both auditory and vestibular structures. This shared developmental origin explains why vestibular dysfunction is a common comorbidity in patients with congenital SNHL.

Recent studies indicate that vestibular dysfunction affects a substantial proportion of children with SNHL. Prevalence estimates vary widely depending on diagnostic methods, ranging from approximately 20% to 70%, with higher rates in those with severe or profound hearing loss [4]. This variability has been confirmed in a recent systematic review by Genovese et al. (2024), which highlighted substantial heterogeneity across studies in terms of patient populations, diagnostic criteria, and vestibular testing protocols, as well as the absence of a universally accepted gold standard for vestibular assessment in congenital hearing loss [5]. Some systematic reviews report even broader ranges, suggesting vestibular deficits may occur in 18–96% of children with SNHL and other neurological conditions [6]. These findings demonstrate a strong association between auditory and vestibular dysfunction within the inner ear. Vestibular dysfunction in children with SNHL is increasingly recognized as a significant contributor to developmental delay and reduced quality of life.

Vestibular impairment may have significant consequences for motor development and postural control in children. The vestibular system plays a central role in maintaining balance, spatial orientation, and gaze stabilization through the vestibulo-ocular reflex (VOR) [7]. Dysfunction of this system may therefore cause delayed acquisition of motor milestones, including independent sitting and walking, as well as impaired coordination and balance during childhood.

Despite the high prevalence of vestibular abnormalities in patients with SNHL, vestibular assessment is not routinely included in standard diagnostic protocols. This may result in underdiagnosis of vestibular deficits, particularly among pediatric populations, where symptoms may be subtle or compensated over time. Furthermore, vestibular testing in children presents methodological challenges related to patient cooperation and the limited availability of specialized diagnostic equipment.

Recent advances in vestibular diagnostics have improved the ability to assess vestibular function through different frequency ranges. Modern testing methods, such as the video head impulse test (vHIT), rotational chair testing using sinusoidal harmonic acceleration (SHA), and vestibular-evoked myogenic potentials (VEMPs), allow for a more extensive evaluation of the semicircular canals and otolith organs [8]. These techniques enable quantitative assessment of the vestibulo-ocular reflex and may reveal vestibular deficits that are not detected by traditional caloric testing. However, the diagnostic specificity of cervical vestibular-evoked myogenic potentials (cVEMPs) remains debated, as recent experimental and clinical evidence suggests that normal cVEMP responses do not necessarily exclude otolithic dysfunction and may reflect activation of multiple vestibular end-organs rather than the saccule alone [9,10]. Moreover, existing studies differ considerably in their methodological design, including the selection of vestibular tests, frequency ranges assessed, and inclusion criteria, which limits direct comparison of results across studies [5].

Although a growing number of studies, including several systematic reviews, have investigated vestibular function in patients with congenital sensorineural hearing loss, relatively few have employed a comprehensive, multimodal battery of vestibular tests covering multiple frequency domains within a well-defined case–control design. As a result, the relationship between the severity of hearing loss and vestibular dysfunction remains incompletely understood.

This study aimed to evaluate vestibular function in patients with congenital bilateral SNHL using a combination of modern vestibular tests, including rotational chair testing with sinusoidal harmonic acceleration, the video head impulse test, and caloric testing. Additionally, this study investigated the relationship between hearing loss severity and vestibular function, as well as the potential impact of vestibular dysfunction on motor development in this patient population.

To the best of current knowledge, this study is among the few to evaluate vestibular function in patients with congenital bilateral SNHL using a combination of low-, mid-, and high-frequency vestibular tests within a case–control design.

## 2. Materials and Methods

### 2.1. Study Population

A total of 202 participants aged 7–31 years were included in this study. The study group comprised 102 patients with congenital bilateral SNHL treated at the Audiology and Phoniatrics Clinic in Bielsko-Biała, Poland. In all patients, bilateral hearing loss had been diagnosed within the first year of life using electrophysiological methods, including auditory brainstem response (ABR) and auditory steady-state response (ASSR).

The control group consisted of 100 healthy volunteers with normal hearing who underwent audiological and vestibular examinations. Participants were recruited through an open public invitation, and the control group was matched to the study group by age and sex.

Patients were excluded if hearing loss developed after the perinatal period, if they had undergone cochlear implantation, or if a conductive component of hearing loss was detected during tuning fork tests or wide-band tympanometry. Additional exclusion criteria included lack of cooperation during examination, unreliable test results, or withdrawal of consent.

Control participants were excluded if hearing thresholds exceeded 20 dB HL at frequencies between 0.5 and 4 kHz or 25 dB HL at 6–8 kHz, if spontaneous or induced nystagmus was observed during vestibular examination, if neurological disorders were present, or if abnormalities were detected during Romberg or Unterberger tests, as well as in cases of a type B tympanogram or when a conductive component was identified based on tuning fork tests.

**Figure 1:**
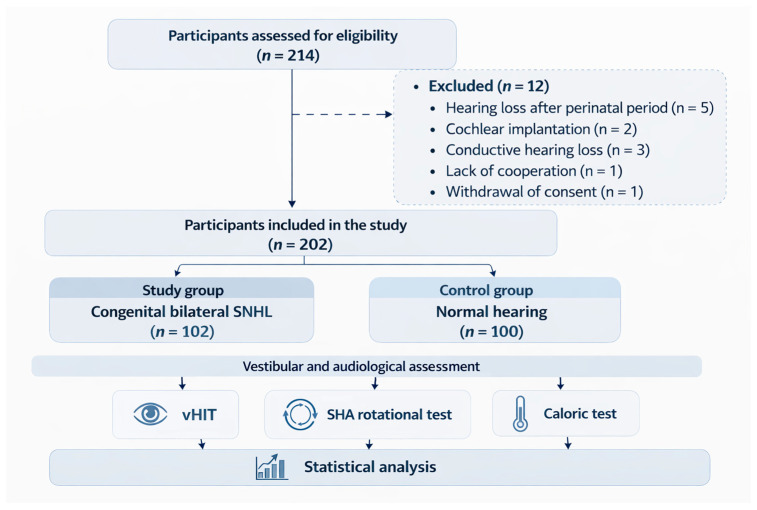
Flow diagram of subject selection and study design.

Children younger than 7 years of age were excluded due to immaturity of oculomotor responses and limited tolerance for vestibular testing procedures. The subject selection process is presented in Figure 1.

A total of 214 individuals were initially assessed for eligibility. After applying the exclusion criteria, 202 participants were included in the study: 102 patients with congenital bilateral sensorineural hearing loss and 100 healthy controls. All participants underwent audiological and vestibular evaluation.

### 2.2. Audiological Assessment

Hearing thresholds were assessed using pure-tone audiometry with an Interacoustics AD629 audiometer, calibrated in accordance with PN-EN 60645-1:2017 [11]. Air-conduction thresholds were measured using TDH-39 headphones at frequencies from 0.5 to 8 kHz.

The severity of hearing loss was determined using the arithmetic mean of hearing thresholds at 0.5, 1.0, 2.0, and 4.0 kHz in the better-hearing ear according to modified World Health Organization criteria [12,13]. Patients were classified into the following categories:

- Mild hearing loss (25–40 dB HL);
- Moderate hearing loss (45–60 dB HL);
- Severe hearing loss (65–85 dB HL);
- Profound hearing loss (≥90 dB HL).

### 2.3. Vestibular Testing

Vestibular function was assessed using videonystagmography (VNG) during sinusoidal harmonic acceleration (SHA) and caloric testing, and with the video head impulse test (vHIT). Vestibular tests were performed in a standardized sequence to provide patient comfort and minimize potential interactions between procedures. The examination began with vHIT, followed by SHA, while caloric testing was performed last due to its greater potential to induce vestibular symptoms. Short rest intervals were provided between tests when required to reduce fatigue and maintain reliability of the recorded responses [14].

#### 2.3.1. Video Head Impulse Test (vHIT)

The video head impulse test was used to evaluate high-frequency vestibular function of the semicircular canals. During the examination, participants wore lightweight goggles equipped with a high-speed camera that recorded eye movements as the examiner applied rapid, unpredictable head impulses.

Head impulses were delivered manually by an experienced examiner in the plane of the horizontal semicircular canals. The VOR gain was calculated automatically by the recording software, and the presence of corrective saccades was also evaluated.

#### 2.3.2. Sinusoidal Harmonic Acceleration (SHA)

Rotational chair testing was performed using sinusoidal harmonic acceleration stimulation to evaluate semicircular canal function. During the test, participants were seated in a motorized rotational chair with their heads stabilized to minimize voluntary movement. The chair rotated sinusoidally at frequencies between 0.04 and 0.64 Hz, producing controlled angular acceleration of the head.

**Figure 2:**
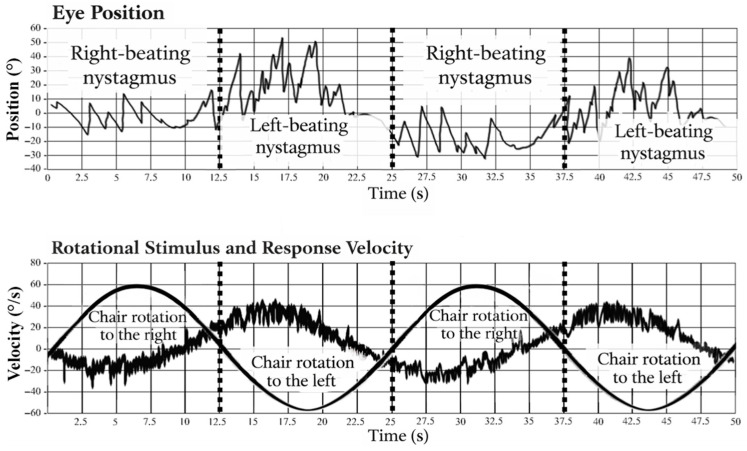
Relationship between chair rotation and nystagmus direction during SHA testing.

Eye movements were recorded using videonystagmography, allowing quantitative assessment of the vestibulo-ocular reflex (VOR). The primary parameter analyzed was VOR gain, defined as the ratio between eye velocity and head velocity during rotational stimulation (Figure 2).

The upper panel shows eye position recordings with right- and left-beating nystagmus defined according to the direction of the fast phase. The lower panel shows the sinusoidal rotational stimulus corresponding to chair rotation to the right and left, along with the resulting vestibulo-ocular reflex response.

Rotational chair testing using sinusoidal harmonic acceleration (SHA) evaluates the vestibulo-ocular reflex as a combined response of both labyrinths and does not allow for reliable side-specific assessment. The obtained parameters therefore reflect global vestibular system function across a range of frequencies rather than separate left- and right-sided responses.

#### 2.3.3. Caloric Test

Caloric testing was performed according to the Fitzgerald–Hallpike protocol using warm and cold air stimulation (30 °C and 44 °C) of the external auditory canal. Each ear was irrigated sequentially while eye movements were recorded using videonystagmography.

The induced nystagmus responses were analyzed by measuring slow-phase velocity (SPV). The results were used to assess vestibular function and compare responses between the right and left labyrinth.

### 2.4. Assessment of Motor Development

Medical records were reviewed to determine the age at which participants achieved independent walking. Attention was paid to potential neurological disorders and to known risk factors for congenital hearing loss, including inherited mutations and intrauterine infections.

### 2.5. Statistical Analysis

Statistical analysis was performed using standard statistical methods. Qualitative variables were presented as counts and percentages. Comparisons between the study and control groups were performed using Pearson’s chi-square test, Fisher’s exact test, or the Fisher–Freeman–Halton test where appropriate. A *p*-value of less than 0.05 was considered statistically significant.

Statistical analyses were performed using PS IMAGO PRO 9.0. Normality of distribution was assessed using the Shapiro–Wilk test. For quantitative variables, comparisons between groups were performed using the Mann–Whitney U test or Kruskal–Wallis test as appropriate. Correlations between hearing loss severity and vestibular parameters were assessed using Spearman’s rank correlation coefficient.

### 2.6. Ethical Approval

The study was conducted in accordance with the principles of the Declaration of Helsinki. Ethical approval was obtained from the Bioethics Committee of the Beskid Medical Chamber in Bielsko-Biała, Poland. Written informed consent was obtained from all adult participants and from the parents or legal guardians of minor participants before inclusion in the study. Participation was voluntary, and all collected data were de-identified to ensure confidentiality.

## 3. Results

### 3.1. Study Population

The study included 202 participants aged 7–31 years. The study group consisted of 102 patients with congenital bilateral sensorineural hearing loss (SNHL), while the control group comprised 100 healthy individuals with normal hearing. The groups were comparable in terms of age and sex distribution.

**Figure 3:**
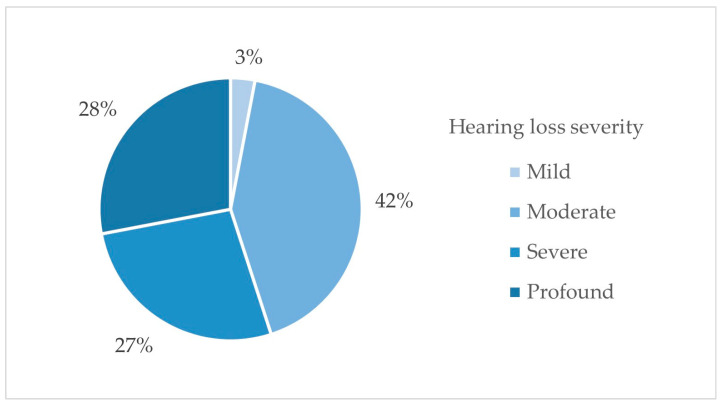
Distribution of hearing loss severity in the study group.

The severity of hearing loss in the study group ranged from moderate to profound according to World Health Organization criteria. The distribution of hearing loss severity is presented in Figure 3.

In the subgroup analysis, patients with mild hearing loss were excluded due to insufficient sample size. The obtained values are presented in Table 1.

### 3.2. Vestibular Function: Comparison Between Groups

#### 3.2.1. Sinusoidal Harmonic Acceleration (SHA)

**Figure 4:**
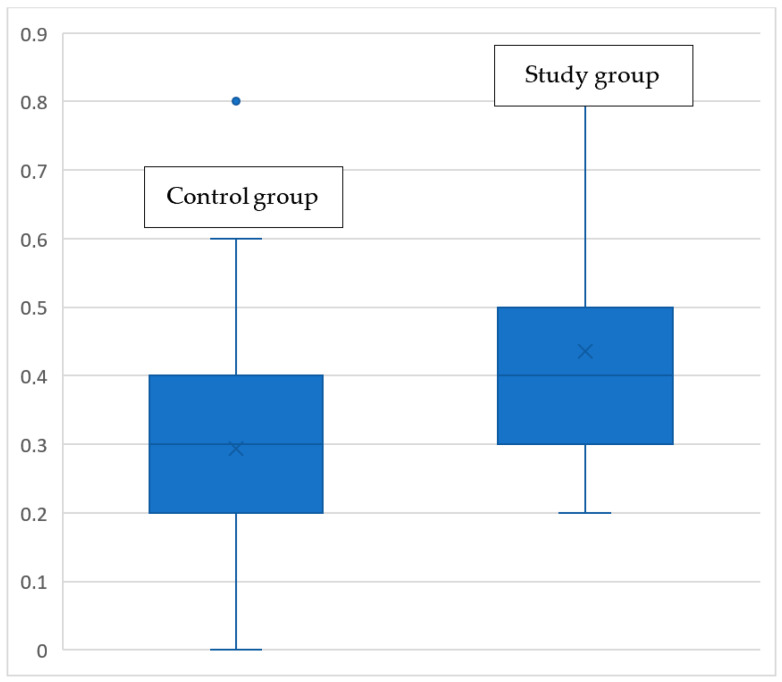
Comparison of the SHA VOR gain between the SNHL and the control group.

The most pronounced differences between groups were observed in the sinusoidal harmonic acceleration (SHA) test. Patients with congenital bilateral SNHL demonstrated significantly lower vestibulo-ocular reflex (VOR) gain than the control group. The distribution of VOR gain values in both groups is shown in Figure 4.

Further analysis indicated that the severity of hearing loss strongly influenced VOR values. Patients with more advanced hearing loss exhibited lower VOR gains on the SHA test. These findings present a clinically relevant reduction in vestibular function associated with increasing severity of hearing loss.

#### 3.2.2. Video Head Impulse Test (vHIT)

Corrective saccades were observed exclusively in the SNHL group and were not detected in the control group. Saccadic eye movements in the vHIT test were recorded in 18.6% of patients with moderate hearing loss, 13.7% of those with severe hearing loss, and 11.1% of those with profound hearing loss. No saccades were observed in patients with mild hearing loss. Additionally, no significant differences were found in the direction of testing.

#### 3.2.3. Caloric Test

Caloric testing did not reveal statistically significant differences between the study and control groups in the analyzed parameters, including unilateral weakness and directional preponderance. The overall distribution of caloric responses was comparable between groups.

### 3.3. Motor Development

The age of independent walking was significantly delayed in patients with profound sensorineural hearing loss compared to the other groups. In this group, the median age was 15 months, whereas in the remaining groups it was 12 months.

### 3.4. Neurological Disorders

Neurological disorders were observed only in patients with moderate, severe, and profound hearing loss. Individuals with mild hearing loss and those in the control group had never been diagnosed with neurological conditions. The reasons for neurological consultations in the respective groups are presented in Table 2.

### 3.5. Genetic Disorders

A total of 7 individuals in the study group were confirmed to carry a genetic mutation associated with congenital hearing loss; 6 are currently undergoing genetic testing, and 2 did not provide genetic test results. Genetic testing was not performed in healthy individuals from the control group. Table 3 summarizes the confirmed mutation types in both groups, stratified by hearing loss severity. The most common mutation identified in the study group was in the GJB2 gene.

### 3.6. Risk Factors for Hearing Loss and Vestibular Dysfunction in the Studied Groups

Analysis of medical histories enabled assessment of risk factors for hearing loss and vestibular dysfunction in patients. The results are presented in Table 4. Risk factors were identified in 35 patients (17.3% of the patients studied).

The analysis of risk factors for hearing loss showed that the most common was maternal diabetes during pregnancy. A documented association was identified in 23 patients (11.4% of all patients), including 14 individuals from the study group with congenital bilateral sensorineural hearing loss. The next most frequent risk factors were prematurity (<37 weeks of gestation) and birth weight below 2500 g (7.9% and 6.4% of all patients, respectively). The least frequent factors included exposure to ototoxic medications, both in mothers during pregnancy and in patients before the age of 1 year, as well as congenital anomalies of the head and neck.

## 4. Discussion

The present study evaluated vestibular function in patients with congenital bilateral sensorineural hearing loss (SNHL) using a comprehensive battery of vestibular tests, including sinusoidal harmonic acceleration (SHA), the video head impulse test (vHIT), and caloric testing. The findings demonstrated a significantly reduced vestibulo-ocular reflex (VOR) gain on the SHA test among patients with SNHL compared with healthy controls. Moreover, lower VOR values were associated with greater hearing loss severity. Although no significant differences were observed in caloric responses or vHIT gain values, corrective saccades during vHIT were detected exclusively in the SNHL group [15]. It should also be noted that SHA reflects bilateral vestibular function and does not provide side-specific information, which may limit direct comparison with tests such as caloric stimulation. Additionally, patients with congenital hearing loss exhibited a delayed age of independent walking, suggesting a functional consequence of vestibular impairment [16,17].

These results support the concept that congenital bilateral SNHL is frequently accompanied by vestibular dysfunction. Previous studies have reported a wide range of prevalence estimates for vestibular abnormalities in this population, from approximately 20% to 70%, with higher rates observed in patients with severe or profound hearing loss. The present findings are consistent with these, as vestibular deficits were more pronounced with increasing severity of hearing loss [8,18]. These observations are consistent with findings from a recent systematic review by Genovese et al. (2024), which emphasized the lack of standardization in vestibular assessment protocols and the resulting variability in reported outcomes. The authors also noted that different vestibular tests evaluate distinct components of the vestibular system and operate across different frequency ranges, which may contribute to inconsistencies between studies [5].

The reduction in VOR gain observed in the SHA test illustrates the high sensitivity of rotational chair testing for detecting bilateral vestibular dysfunction. Unlike caloric testing, which assesses vestibular responses at very low frequencies, SHA evaluates function across a wider and more physiological frequency range. This may explain why abnormalities were detected in SHA despite the absence of significant differences in caloric responses. These findings support the notion that different vestibular tests assess distinct aspects of vestibular function and should be interpreted in a complementary manner [14,19].

Although vHIT gain values did not vary markedly between groups, corrective saccades were observed exclusively in patients with SNHL. These saccades are considered a marker of vestibular dysfunction, even when VOR gain remains within the normal range, and may reflect compensatory mechanisms that maintain gaze stability [20]. This observation suggests that subtle vestibular deficits may be present despite preserved quantitative parameters, pointing out the importance of qualitative analysis in vHIT interpretation.

The delayed age of independent walking observed in patients with SNHL further supports the functional relevance of vestibular impairment. The vestibular system plays a key role in postural control and motor development, and its dysfunction may contribute to delayed motor milestone acquisition. These outcomes are consistent with previous reports indicating delayed motor development in children with congenital hearing loss [21].

From a clinical perspective, vestibular dysfunction in patients with SNHL remains underdiagnosed, as vestibular assessment is not routinely included in standard diagnostic protocols [18]. Early identification of vestibular deficits may facilitate the timely implementation of rehabilitation strategies to improve balance and motor development [22]. However, based on the present findings, routine vestibular screening for all children with SNHL may not be justified, and further studies are required to establish optimal screening strategies [23].

Several limitations of this study should be considered. Vestibular testing is inherently variable and less standardized than audiological assessment, which may affect measurement accuracy [14,19,24]. Responses may also be influenced by anatomical variability, technical factors related to eye movement recording, and patient cooperation [25]. The lack of systematic radiological evaluation (e.g., temporal bone CT or MRI) is a limitation of this study, as structural abnormalities of the inner ear may influence vestibular function. In addition, due to the predominantly symmetrical nature of hearing loss in the study population, comparisons between better- and worse-hearing ears were limited, which may have reduced the ability to detect potential side-specific relationships between hearing loss and vestibular function. Another limitation of the present study is that classification based on the better-hearing ear did not allow for detailed analysis of asymmetrical hearing loss configurations or interaural differences, which may influence vestibular function and its clinical presentation. In addition, the exclusion of children younger than seven years and the cross-sectional design limit the generalizability of the findings and preclude assessment of longitudinal changes in vestibular function [26].

The strengths of this study include a relatively large sample size, a well-matched control group, and the use of multiple complementary vestibular tests that assess different frequency domains of vestibular function. Furthermore, all examinations were performed according to standardized protocols, thereby increasing the reliability of the results.

Importantly, no single vestibular test is currently considered a gold standard for assessing vestibular dysfunction in patients with congenital hearing loss, further supporting the need for a multimodal diagnostic approach [5]. These observations are consistent with recent systematic evidence highlighting the lack of standardization and variability in vestibular assessment protocols across studies. Future studies ought to focus on longitudinal evaluation of vestibular function in patients with congenital hearing loss and on defining optimal strategies for vestibular screening and rehabilitation in this population.

## 5. Conclusions

The results of this study demonstrate that congenital bilateral sensorineural hearing loss is frequently associated with vestibular dysfunction. Patients with SNHL showed significantly reduced vestibulo-ocular reflex values during sinusoidal harmonic acceleration testing, and the severity of hearing loss was associated with greater vestibular impairment.

Although caloric responses and vHIT VOR gain values did not differ significantly between groups, the presence of corrective saccades during vHIT and the delayed onset of independent walking suggest clinically relevant vestibular involvement in this population.

These outcomes indicate that the rotational chair testing may be particularly useful for detecting vestibular dysfunction in patients with congenital SNHL. Comprehensive vestibular assessment may be considered in selected patients with congenital hearing loss, particularly in the presence of motor developmental delays or balance-related symptoms.

## Figures and Tables

**Table 1:** Baseline characteristics of the study and control groups.

			Control Group *n* = 100	Moderate Hearing Loss *n* = 43	Severe Hearing Loss *n* = 29	Profound Hearing Loss *n* = 27	*p*-Value	
Age [years]			15 (10; 21.75)	14 (10; 18)	13 (10.5; 23)	15 (11; 24)	0.72	
Sex		Female	46 (46%)	19 (44.2%)	17 (58.6%)	11 (40.7%)	0.54	
		Male	54 (54%)	24 (55.8%)	12 (41.4%)	16 (59.3%)		
Better-hearing ear		Right	22 (22%)	13 (30.2%)	6 (20.7%)	9 (33.3%)	0.51	<0.001
		Left	13 (13%)	19 (44.2%)	15 (51.7%)	14 (51.9%)	<0.001	
		Same hearing threshold	65 (65%)	11 (25.6%)	8 (27.6%)	4 (14.8%)	<0.001	
Hearing loss [dB]			15 (10; 20)	60 (55; 60)	90 (80; 90)	110 (100; 120)	<0.001	
Tympanogram type for right ear		A	100 (100%)	43 (100%)	23 (79.3%)	26 (96.3%)	<0.001	<0.001
		B	0 (0%)	0 (0%)	0 (0%)	0 (0%)	-	
		C	0 (0%)	0 (0%)	6 (20.7%)	1 (3.7%)	<0.001	
Tympanogram type for the left ear		A	100 (100%)	43 (100%)	23 (79.3%)	26 (96.3%)	<0.001	<0.001
		B	0 (0%)	0 (0%)	0 (0%)	0 (0%)	-	
		C	0 (0%)	0 (0%)	6 (20.7%)	1 (3.7%)	<0.001	
VOR gain value in the SHA test			0.4 (0.3; 0.5)	0.3 (0.2; 0.5)	0.2 (0.1; 0.4)	0.2 (0.1; 0.3)	<0.001	
Unilateral Weakness in caloric test [%]			14 (7; 23)	11 (6; 20)	14 (6.5; 26.5)	14 (6; 20)	0.54	
Directional Preponderance in caloric test [%]			13 (6; 19.5)	10 (4; 17)	12 (7.5; 17)	13 (7; 22)	0.28	
Total response—right ear in caloric test [°/s]			22.18 (14.88; 31.6)	28.54 (15.61; 37.13)	28.22 (17.99; 39.75)	30.05 (24.64; 33.52)	0.051	
Total response—left ear in caloric test [°/s]			29.62 (16.5; 40.13)	26.3 (15.78; 31.46)	29.83 (16.01; 37.04)	34.53 (20.05; 43.82)	0.06	
VOR gain in vHIT for the right side			0.96 (0.845; 1.073)	0.95 (0.85; 1.05)	0.95 (0.835; 1.05)	0.95 (0.88; 1.03)	0.92	
VOR gain in vHIT for the left side			1 (0.888; 1.07)	0.99 (0.95; 1.11)	0.95 (0.78; 1.115)	1.07 (0.89; 1.15)	0.23	
Saccades	None		100 (100%)	35 (81.4%)	25 (86.2%)	24 (88.9%)	<0.001	<0.001
	Rightward		0 (0%)	5 (11.6%)	2 (6.9%)	0 (0%)	0.002	
	Leftward		0 (0%)	0 (0%)	1 (3.4%)	1 (3.7%)	0.077	
	Bilateral		0 (0%)	3 (7%)	1 (3.4%)	2 (7.4%)	0.019	
Age of independent walking [months]			12 (12; 12)	12 (12; 15)	12 (11; 14.5)	15 (12; 18)	<0.001	
Neurological disorders			0 (0%)	11 (25.6%)	11 (37.9%)	9 (33.3%)	<0.001	
Genetic disorders (confirmed and under diagnostic evaluation)			0 (0%)	6 (14%)	6 (20.7%)	3 (11.1%)	<0.001	
Genetic disorders (confirmed only)			0 (0%)	4 (9.3%)	1 (3.4%)	2 (7.4%)	0.002	

**Table 2:** Reasons for neurological consultations in the study group.

Hearing Loss Severity		Mild Hearing Loss *n* = 3	Moderate Hearing Loss *n* = 43	Severe Hearing Loss *n* = 29	Profound Hearing Loss *n* = 27	Total
The number of neurologically evaluated patients		0 (0%)	11 (25.6%)	11 (37.9%)	9 (33.3%)	31
Indications for consultation	Hypotonia	0	9	8	8	25
	Hypertonia	0	1	1	1	3
	Other	0	migraine with aura (*n* = 1)	epilepsy (*n* = 1)	vertigo (*n* = 1)	6
			Parry–Romberg syndrome (*n* = 1)	Impaired balance up to 2 years of age (*n* = 1)	Antley–Bixler syndrome (*n* = 1)	

**Table 3:** Summary of genetic diagnostic results in patients from the study group stratified by hearing loss severity.

Severity of Hearing Loss	Mild Hearing Loss	Moderate Hearing Loss	Severe Hearing Loss	Profound Hearing Loss
Confirmed mutations	none	➢ 9p22 deletion	➢ c.35delG in GJB2 gene	➢ mutation p.Glu18 7
		➢ p.L90P in GJB2 gene		➢ 10q26 in the FGFR2 gene (Antley-Bixler syndrome)
		➢ 6p21.32 in COL11A2 gene (Stickler syndrome type 3)		
		➢ c.362dupC in FKBP14 gene		
		➢ p.E120del in GJB2 gene		
Number of patients undergoing genetic testing	0	1	5	0
Number of patients without available test results	0	1	0	1

**Table 4:** Frequency of specific risk factors for hearing loss across groups stratified by hearing loss severity.

Risk Factors	Control Group *n* = 100	Mild Hearing Loss *n* = 3	Moderate Hearing Loss *n* = 43	Severe Hearing Loss *n* = 29	Profound Hearing Loss *n* = 27	Total
Number of patients with confirmed risk factors	9 (9%)	1 (33.3%)	9 (20.9%)	8 (27.6%)	8 (29.6%)	35 (100%)
Genetically confirmed mutation	0	0	4	1	2	7 (20%)
confirmed congenital TORCH infection	0	1	2	4	2	9 (25.7%)
Maternal diabetes during pregnancy	9	0	5	5	4	23 (65.7%)
Congenital anomaly of the head or neck	0	0	1	0	1	2 (5.7%)
Exposure to ototoxic drugs	0	0	0	1	0	1 (2.9%)
jaundice requiring exchange transfusion	1	0	2	4	3	10 (28.6%)
Prematurity (<37 weeks of gestation)	3	1	4	5	3	16 (45.7%)
Low birth weight (<2500 g)	2	1	4	3	3	13 (37.1%)

## Data Availability

The data presented in this study are available on reasonable request from the corresponding author. The data are not publicly available due to privacy and ethical principles restrictions.

## Abbreviations

The following abbreviations are used in this manuscript:

SNHL	sensorineural hearing loss
VOR	vestibulo-ocular reflex
vHIT	video head impulse test
SHA	sinusoidal harmonic acceleration
VEMP	vestibular-evoked myogenic potentials
cVEMP	cervical vestibular-evoked myogenic potentials
